# Prognostic Indicators of Preoperative Fluorouracil, Leucovorin, Oxaliplatin, and Docetaxel Efficacy in Locally Advanced Gastroesophageal and Gastric Cancer: Integrating Biomarker Analysis and Clinicopathological Factors

**DOI:** 10.1200/PO-24-00925

**Published:** 2025-08-01

**Authors:** Amane Jubashi, Izuma Nakayama, Naoya Sakamoto, Shogo Takei, Yuki Matsubara, Yu Miyashita, Seiya Sato, Shinpei Ushiyama, Akinori Kobayashi, Ukyo Okazaki, Dai Okemoto, Kazumasa Yamamoto, Saori Mishima, Daisuke Kotani, Akihito Kawazoe, Tadayoshi Hashimoto, Yoshiaki Nakamura, Yasutoshi Kuboki, Hideaki Bando, Takashi Kojima, Takayuki Yoshino, Takeshi Kuwata, Kazuma Sato, Takeo Fujita, Mitsumasa Yoshida, Masahiro Yura, Takahiro Kinoshita, Kohei Shitara

**Affiliations:** ^1^ Department of Gastroenterology and Gastrointestinal Oncology, National Cancer Center Hospital East, Kashiwa, Japan; ^2^ Department of Gastroenterology and Hepatology, Nagasaki University Graduate School of Biomedical Sciences, Nagasaki, Japan; ^3^ Department of Pathology and Clinical Laboratories, National Cancer Center Hospital East, Kashiwa, Japan; ^4^ Division of Pathology, Exploratory Oncology Research & Clinical Trial Center, National Cancer Center, Kashiwa, Japan; ^5^ Translational Research Support Office, National Cancer Center Hospital East, Kashiwa, Japan; ^6^ Department of Experimental Therapeutics, National Cancer Center Hospital East, Kashiwa, Japan; ^7^ Department of Esophageal Surgery, National Cancer Center Hospital East, Kashiwa, Japan; ^8^ Department of Gastric Surgery, National Cancer Center Hospital East, Kashiwa, Japan

## Abstract

**PURPOSE:**

Perioperative fluorouracil, leucovorin, oxaliplatin, and docetaxel (FLOT) is a standard treatment for locally advanced gastric/gastroesophageal junction cancer (GC/GEJC). The impact of biomarker status on the efficacy of perioperative FLOT remains unclear. This study evaluated the association between clinicopathological features, including biomarker status, and the efficacy of perioperative FLOT in patients with resectable GC/GEJC.

**PATIENTS AND METHODS:**

A retrospective observational study was conducted by reviewing medical records of patients treated with perioperative FLOT between February 2020 and March 2024. Eligible patients had histologically confirmed adenocarcinoma, resectable disease at stages cT2-4a and/or N0-3, M0, and underwent biomarker testing.

**RESULTS:**

Among 116 eligible patients, human epidermal growth factor receptor 2 (HER2) positivity was observed in 7.8%, whereas PD-L1 combined positive score (CPS) of ≥1, ≥5, and ≥10 was detected in 90.5%, 44.0%, and 15.5% of patients, respectively. Claudin-18 isoform 2 (CLDN18.2) positivity (2+/3+ in ≥75% of tumor cells) was observed in 30.2% of patients. Major pathological response (MPR) and pathological complete response (pCR) rates were 22.4% (95% CI, 15.3 to 31.0) and 8.6% (95% CI, 4.2 to 15.3), respectively. Diffuse-type histology was a negative indicator for pathological response. CLDN18.2 expression decreased significantly after preoperative FLOT, with the median H-score declining from 285.0 to 187.5 (*P* < .001) in patients with CLDN18.2 positivity at initiation. Maintained CLDN18.2 positivity was more frequently observed in patients without MPR compared with those with MPR (53.8% *v* 12.5%, *P* = .05).

**CONCLUSION:**

HER2, PD-L1, and CLDN18.2 statuses were not linked to pathological response to FLOT in resectable GC/GEJC. CLDN18.2 expression significantly decreased after preoperative FLOT but remained higher in patients without MPR, suggesting that CLDN18.2-targeted therapy may warrant investigation in the perioperative setting.

## INTRODUCTION

Curative gastrectomy with chemotherapy is the standard of care for locally advanced gastric or gastroesophageal junction cancer (GC/GEJC). In Europe and North America, perioperative fluorouracil, leucovorin, oxaliplatin, and docetaxel (FLOT) has become the preferred approach.^1^ Although upfront surgery followed by adjuvant chemotherapy has traditionally been more common in Asia,^2-4^ recent trials have demonstrated the benefits of perioperative chemotherapy in the Asian population.^5,6^ Previously, we reported the feasibility of perioperative FLOT in Japanese patients with locally advanced GC/GEJC.^7^

**CONTEXT**

**Key Objective**
We analyzed the associations between clinicopathological features, biomarker status, and the efficacy of perioperative fluorouracil, leucovorin, oxaliplatin, and docetaxel (FLOT) in patients with resectable, locally advanced gastric/gastroesophageal junction cancer (GC/GEJC).
**Knowledge Generated**
Pathological response to preoperative FLOT therapy was not significantly influenced by human epidermal growth factor receptor 2, PD-L1, or claudin-18 isoform 2 (CLDN18.2) status. Following preoperative FLOT, CLDN18.2 expression levels decreased, with 55.9% of cases converting from positive to negative status. Moreover, CLDN18.2 expression was comparably lower in patients achieving major pathological response (MPR) compared with those without MPR.
**Relevance**
CLDN18.2-targeted therapy may represent a potential therapeutic approach worth investigating in patients who do not respond to preoperative FLOT.

The randomized phase III MATTERHORN study showed that adding durvalumab, an anti–PD-L1 antibody, to FLOT significantly improves pathological complete response (pCR) rates compared with FLOT alone.^8^ Targeted therapies and immunotherapy based on molecular profiles, including human epidermal growth factor receptor 2 (HER2), mismatch repair (MMR), PD-L1, and claudin-18 isoform 2 (CLDN18.2), have significantly prolonged the survival of patients with metastatic GC/GEJC.^9-16^ Although not yet standard of care in resectable disease, biomarker-driven approaches show promise, with anti-HER2 therapies demonstrating improved pathological responses in HER2-positive cases.^17,18^ For immunotherapy, patient selection based on PD-L1 expression and MMR status appears critical.^19,20^ Although several studies have examined HER2 and PD-L1 biomarkers in neoadjuvant settings,^21-24^ data on CLDN18.2 expression and treatment outcomes remain limited.

In this study, we analyzed the associations between clinicopathological features, biomarker status, and the efficacy of perioperative FLOT in patients with resectable, locally advanced GC/GEJC.

## PATIENTS AND METHODS

### Study Design and Patient Selection

This retrospective observational study was conducted at the National Cancer Hospital East, Kashiwa, Japan. We reviewed the medical records of patients treated with perioperative FLOT at our institution between February 2020 and March 2024. Eligible patients met the following criteria: (1) histologically confirmed adenocarcinoma of locally advanced GC/GEJC; (2) resectable disease at cT2-4a and/or N0-3, M0 according to the eighth edition of the Union for International Cancer Control (UICC) TNM classification; (3) received at least one cycle of FLOT; and (4) completion of biomarker testing, including HER2, MMR, PD-L1, and CLDN18.2. Written informed consent for treatment and biomarker analysis was obtained from all patients (UMIN000019129). The dosing and schedule of FLOT were previously described.^7^

### Molecular Analysis

Biomarker status was prospectively assessed by two board-certified pathologists (N.S. and T.K.) using formalin-fixed paraffin-embedded tissue specimens from primary tumor sites. HER2 status was determined by immunohistochemistry (IHC) with a monoclonal anti-HER2 antibody (PATHWAY HER2 [4B5], Ventana, Tucson, AZ) and fluorescence in situ hybridization (FISH) using the PathVysion HER2 Probe Kit (Abbott Laboratories, Abbott Park, IL). HER2 positivity was defined as IHC 3+ or IHC 2+ with FISH positivity. PD-L1 expression was assessed using anti–PD-L1 monoclonal antibodies (Clone 22C3 or 28-8) and quantified by the combined positive score (CPS), calculated as the number of PD-L1–positive cells (tumor cells, lymphocytes, and macrophages) divided by the total number of tumor cells and multiplied by 100.

MMR status was evaluated by IHC with monoclonal antibodies for MLH1 (ES05), MSH2 (FE11), PMS2 (EP51), and MSH6 (EP49; Agilent Technologies, Santa Clara, CA). Tumors lacking MLH1, MSH2, PMS2, or MSH6 expression were classified as MMR-deficient (dMMR), whereas those retaining the expression of all four proteins were categorized as MMR-proficient (pMMR). CLDN18.2 expression was assessed by IHC using a CLDN18 antibody (Clone 43-14A, Roche Ventana, Oro Valley, AZ). CLDN18.2 positivity was defined as moderate (2+) to strong (3+) IHC staining in ≥75% of tumor cells. Epstein-Barr virus (EBV) status was determined by chromogenic in situ hybridization for EBV-encoded RNA (EBER) with fluorescein-labeled oligonucleotide probes (INFORM EBER Probe, Ventana).

### Assessments

Comprehensive clinicopathological data were collected for each patient, including age, sex, Eastern Cooperative Oncology Group (ECOG) performance status (PS), primary tumor location (GEJ or gastric), clinical T and N stages, Borrmann classification (type 4 or non-type 4), Lauren's classification (intestinal or diffuse), and histology (signet ring cell carcinoma or not). Biomarker status was categorized as HER2 (positive or negative), PD-L1 CPS (positive or negative using cutoff values of 1, 5, and 10), MMR (deficient or proficient), EBV (positive or negative), and CLDN18.2 (IHC staining intensity 2+/3+ in ≥25%, 40%, or 75% of tumor cells). For CLDN18.2 expression, the H-score was calculated using the following formula: 3 × percentage of strongly stained cells (3+) + 2 × percentage of moderately stained cells (2+) + 1 × percentage of weakly stained cells (1+). In patients with CLDN18.2-positive tumors before administration of FLOT, we evaluated changes in CLDN18.2 expression between baseline and surgical specimens after preoperative FLOT using both H-score and percentage of tumor cells with ≥2+ staining intensity.

### Histopathological Analysis

Histopathological observation was assessed according to the Japanese Gastric Cancer Association (JGCA) criteria: grade 0 (no effect), grade 1a (very slight effect, >2/3 of tumor viable), grade 1b (slight effect, 1/3 to 2/3 of tumor viable), grade 2a (considerable effect, 1/10 to <1/3 of tumor viable), grade 2b (marked effect, <1/10 of tumor viable), and grade 3 (complete response, no viable tumor cells).^25^ Grades 3 and 2b + 3 correspond to pCR and major pathological response (MPR), respectively.

### Statistical Analysis

The proportion of pathological responses was calculated with 95% CI using the Clopper-Pearson method. Categorical variables were compared using Fisher's exact test or chi-square test, whereas continuous variables were compared using two-sample t-test and Mann-Whitney U test. A two-sided *P* value of <.05 was considered statistically significant.

All analyses were conducted using Python 3.10.9 within the Anaconda Navigator environment (version 23.5.2). The following Python libraries were used for data manipulation, analysis, and visualization: NumPy (version 1.23.5), Pandas (version 1.5.3), SciPy (version 1.10.0), Statsmodels (version 0.13.5), Matplotlib (version 3.7.0), and Plotly (version 5.9.0).

### Ethics Approval and Consent to Participate

All procedures were conducted in accordance with the ethical standards of the responsible committee on human experimentation (institutional and national) and with the Helsinki Declaration of 1964 and its later versions. Informed consent or its equivalent was obtained from all patients. This study was approved by the ethics committee of our institution, the National Cancer Center Hospital East Certified Review Board (Approval ID: 2017-120, Approval date: September 26, 2017), and was conducted in accordance with the guidelines for biomedical research as stipulated in the Declaration of Helsinki.

## RESULTS

### Patient Characteristics

**FIG 1.**
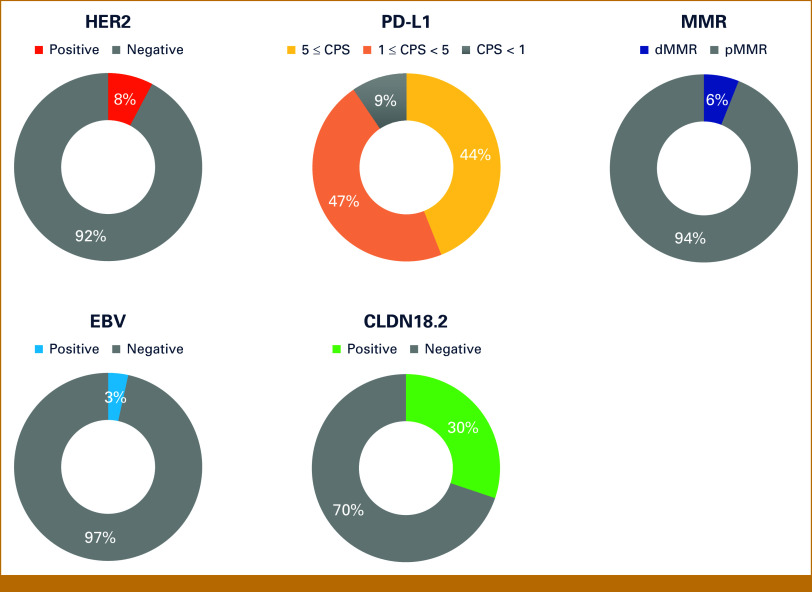
Biomarker analysis results. Distribution of biomarkers in this study. The pie charts show the prevalence of HER2 (positive: 8%, negative: 92%), PD-L1 (CPS ≥ 5: 44%, 1 ≤ CPS < 5: 47%, CPS < 1: 9%), MMR status (dMMR: 6%, pMMR: 94%), EBV (positive: 3%, negative: 97%), and CLDN18.2 (positive: 30%, negative: 70%). CLDN18.2, claudin-18 isoform 2; CPS, combined positive score; dMMR, deficient MMR; EBV, Epstein-Barr Virus; HER2, human epidermal growth factor receptor 2; MMR, mismatch repair; pMMR, proficient MMR.

**FIG A1.**
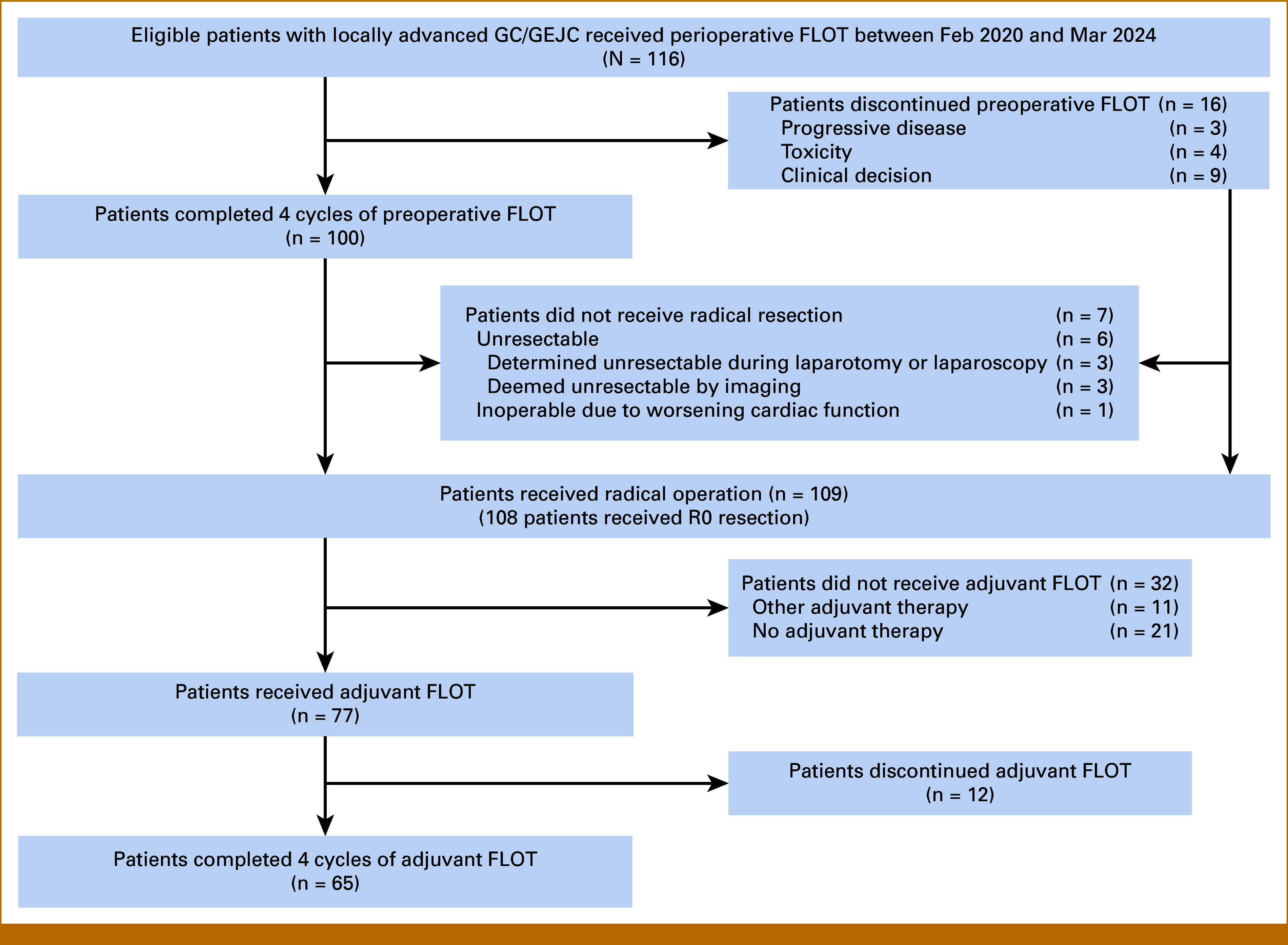
Study profile. This flowchart illustrates the treatment pathway of patients enrolled in this study. FLOT, fluorouracil, leucovorin, oxaliplatin, and docetaxel; GC/GEJC, gastric/gastroesophageal junction cancer.

A total of 116 eligible patients were analyzed (Table 1, Appendix Fig A1). The median age was 70 years (range, 29-84). Forty-nine patients (42.2%) had GEJ primary tumors, and 70 patients (60.3%) had diffuse-type histology. Most patients had T3 or higher and node-positive disease. Biomarker analysis results are shown in Figure 1. HER2 positivity was observed in nine patients (7.8%). PD-L1 CPS was ≥1 in 105 patients (90.5%), ≥5 in 51 patients (44.0%), and ≥10 in 18 patients (15.5%). CLDN18.2 positivity was observed in 35 patients (30.2%). Using cutoff values of 40% and 25% for tumor areas with 2+/3+ IHC staining intensity, CLDN18.2 expression was observed in 65 (56.0%) and 69 patients (59.5%), respectively. In addition, seven patients (6.0%) had dMMR, and four patients (3.4%) were EBV-positive.

**TABLE 1.** Patient Characteristics

Characteristics	N = 116, No. (%)
Age	
Median (range)	70 (29-84)
≥65	81 (68.8)
≥75	32 (27.6)
Sex	
Male	88 (75.9)
Female	28 (24.1)
ECOG PS	
0	99 (85.3)
1	17 (14.7)
Tumor location	
GEJ	49 (42.2)
Gastric	67 (57.8)
Borrmann type	
Type 4	12 (10.3)
Non–type 4	104 (89.7)
Lauren's type	
Diffuse	70 (60.3)
Intestinal	46 (39.7)
Histology	
Sig	20 (17.2)
Non-sig	96 (82.8)
Clinical T	
T1-T2	10 (8.6)
T3	62 (53.4)
T4	44 (37.9)
Clinical N	
N0	9 (7.8)
N1-N3	107 (92.2)

Abbreviations: ECOG PS, Eastern Cooperative Oncology Group Performance Status; GEJ, gastroesophageal junction; Sig, signet ring cell carcinoma.

### Efficacy

Among the 116 patients, 100 patients (86.2%) completed four cycles of preoperative FLOT (Appendix Fig A1). One hundred nine patients (94.0%) underwent radical surgery after FLOT, of which 108 (93.1% [95% CI, 86.9 to 97.0]) achieved R0 resection, including 12 patients who discontinued FLOT before four cycles. Seventy-seven patients (66.4%) received at least one cycle of postoperative FLOT, and 61 patients (52.6%) completed eight cycles of perioperative FLOT (Appendix Fig A1).

MPR and pCR rates were 22.4% (95% CI, 15.3 to 31.0) and 8.6% (95% CI, 4.2 to 15.3), respectively (Table2).

**TABLE 2.** Pathological Response

Histopathological Effect (JGCA)^a^	N = 116, No. (%)
0	1 (0.9)
1a	30 (25.9)
1b	30 (25.9)
2a	22 (19.0)
2b	16 (13.8)
3	10 (8.6)
pCR	10 (8.6)
MPR	26 (22.4)
≥Grade 1b	78 (67.2)

Abbreviations: JCGA, Japanese Gastric Cancer Association; MPR, major pathological response; pCR, pathological complete response.

a Histopathological effect was assessed according to Japanese Classification of Gastric Carcinoma 15th. Grade 0, tumor unaffected; grade 1a, <1/3 affected; grade 1b, 1/3-2/3 affected; grade 2a, 1/10-2/3 to entirely affected; grade 2b, <1/10 viable tumor cells; grade 3, no residual tumor. Grades 3 and 2b + 3 correspond to pCR and MPR, respectively.

### Association Between Clinicopathological Factors and Pathological Response

**FIG 2.**
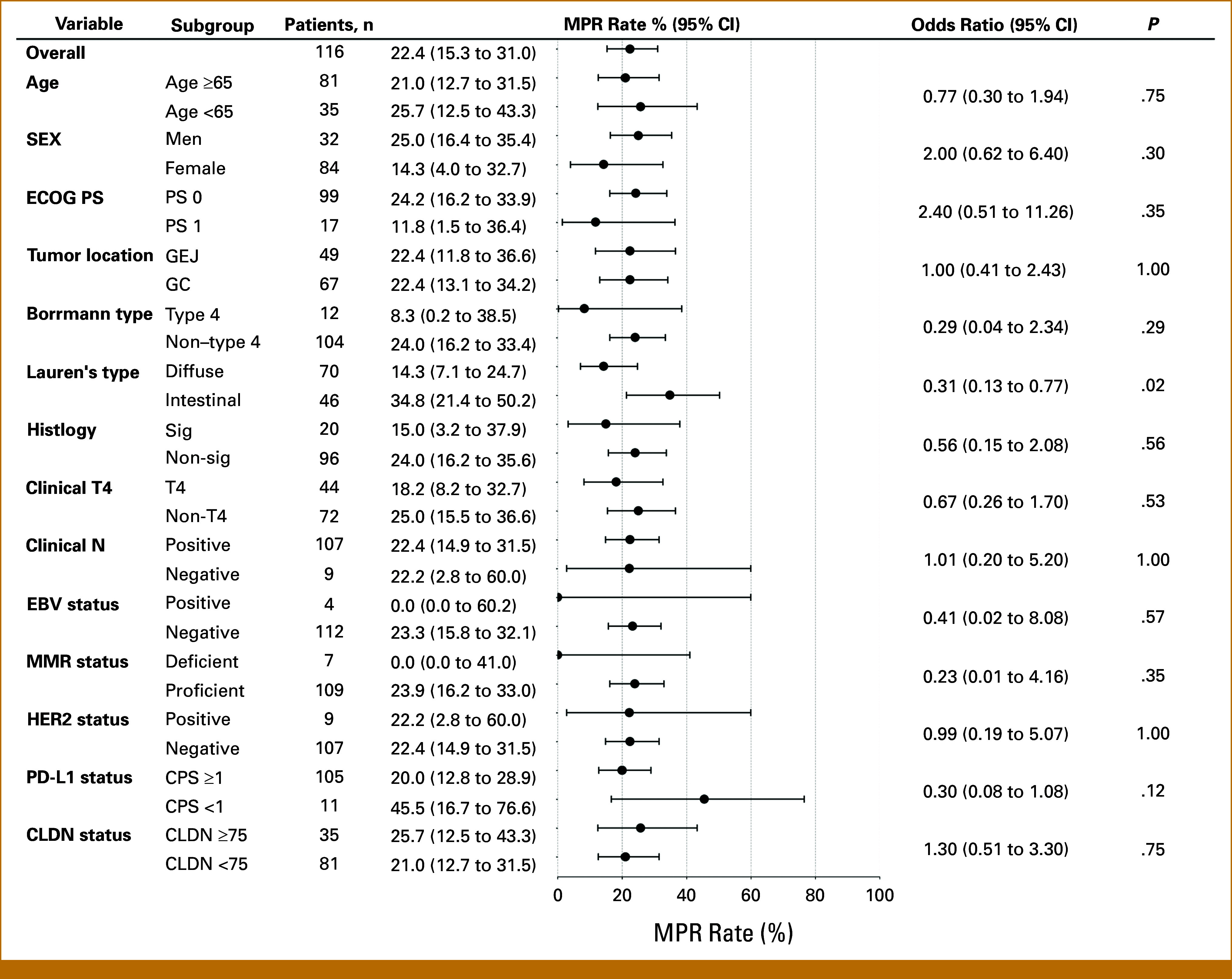
MPR by subgroup. Forest plot depicting MPR rates across various subgroups. The plot shows MPR rates with 95% CI for each subgroup, along with the corresponding odds ratios (95% CI) and *P* values. Lauren's type demonstrates a statistically significant difference in MPR rates between the diffuse and intestinal types (*P* = .02). CPS, combined positive score; EBV, Epstein-Barr virus; ECOG PS, Eastern Cooperative Oncology Group Performance Status; GC/GEJC, gastric/gastroesophageal junction cancer; MPR, major pathological response; HER2, human epidermal growth factor receptor 2; MMR, mismatch repair.

The analysis results of the association between MPR and clinicopathological factors are summarized in Figure 2. Univariable analysis revealed that the diffuse type had significantly lower MPR compared with the intestinal type (odds ratio [OR], 0.31 [95% CI, 0.13 to 0.77]; *P* = .02). dMMR showed poor pathological response, with none of the seven cases achieving. Similarly, none of the EBV-positive cases achieved MPR. HER2 positivity, PD-L1 CPS (≥1, 5, and 10), and CLDN18.2 expression with 2+/3+ staining intensity (≥25, 40%, and 75%) were not significantly associated with MPR (Appendix Table A1).

### Change in CLDN18.2 Expression Following Preoperative FLOT

**FIG 3.**
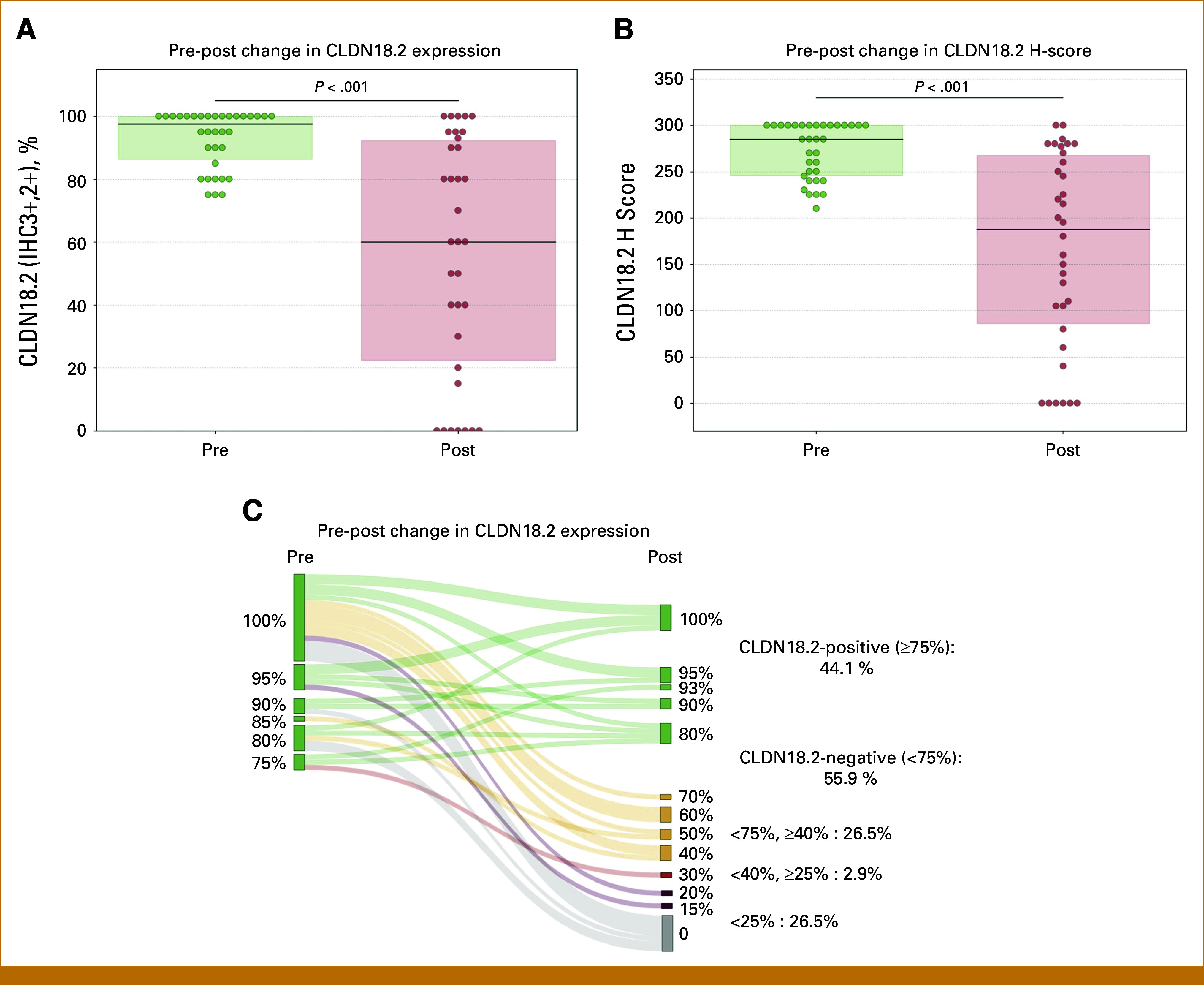
Changes in CLDN18.2 expression before and after treatment. (A) Bee-swarm plot showing the distribution of CLDN18.2 expression rates (IHC staining intensity 2+/3+). The median expression rate significantly decreased from 95.0% (IQR, 87.5%-100.0%) before treatment to 60.0% (IQR, 22.5%-92.2%) after treatment (*P* < .001). (B) Bee-swarm plot showing the distribution of CLDN18.2 H-scores. The median H-score significantly decreased from 285.0 (IQR, 247.5-300.0) before treatment to 187.5 (IQR, 86.2-267.5) after treatment, with a difference of −97.5 (*P* < .001). (C) Sankey diagram illustrating changes in CLDN18.2 expression between the pre- and post-treatment samples. The diagram shows the distribution of expression levels after treatment in patients with CLDN18.2-positive tumors at baseline. Post-treatment expression levels were stratified into four categories: CLDN18.2-positive (≥75%: 44.1%) and CLDN18.2-negative (<75%: 55.9%), with the latter further subdivided into <75%, ≥40% (26.5%); <40%, ≥25% (2.9%); and <25% (26.5%). Flow widths represent patient proportions, and node colors indicate expression levels (green: ≥75%; orange: <75%, ≥40%; red: <40%, ≥25%; dark red: <25%). CLDN18.2, claudin-18 isoform 2; IHC, immunohistochemistry.

CLDN18.2 expression before and after preoperative FLOT was assessable in 34 patients with CLDN18.2-positive tumors at baseline. Of these, 30 patients (88.2%) completed four cycles of preoperative FLOT. The proportion of CLDN18.2-expressing tumor cells with 2+/3+ staining intensity decreased from a median of 95.0% (IQR, 87.5%-100.0%) at baseline to 60.0% (IQR, 22.5%-92.2%) after preoperative FLOT (*P* < .001; Fig 3A). The median H-score for CLDN18.2 expression declined significantly from 285.0 (IQR, 247.5-300.0) to 187.5 (IQR, 86.2-267.5, *P* < .001; Fig 3B). Among these patients, CLDN18.2 positivity was maintained in 44.1% (n = 15). Using different cutoff values, CLDN18.2-expressing tumor cells with 2+/3+ staining intensity in ≥40% and ≥25% of tumor cells were present in 70.6% and 73.5% of resected specimens, respectively (Fig 3C).

**FIG 4.**
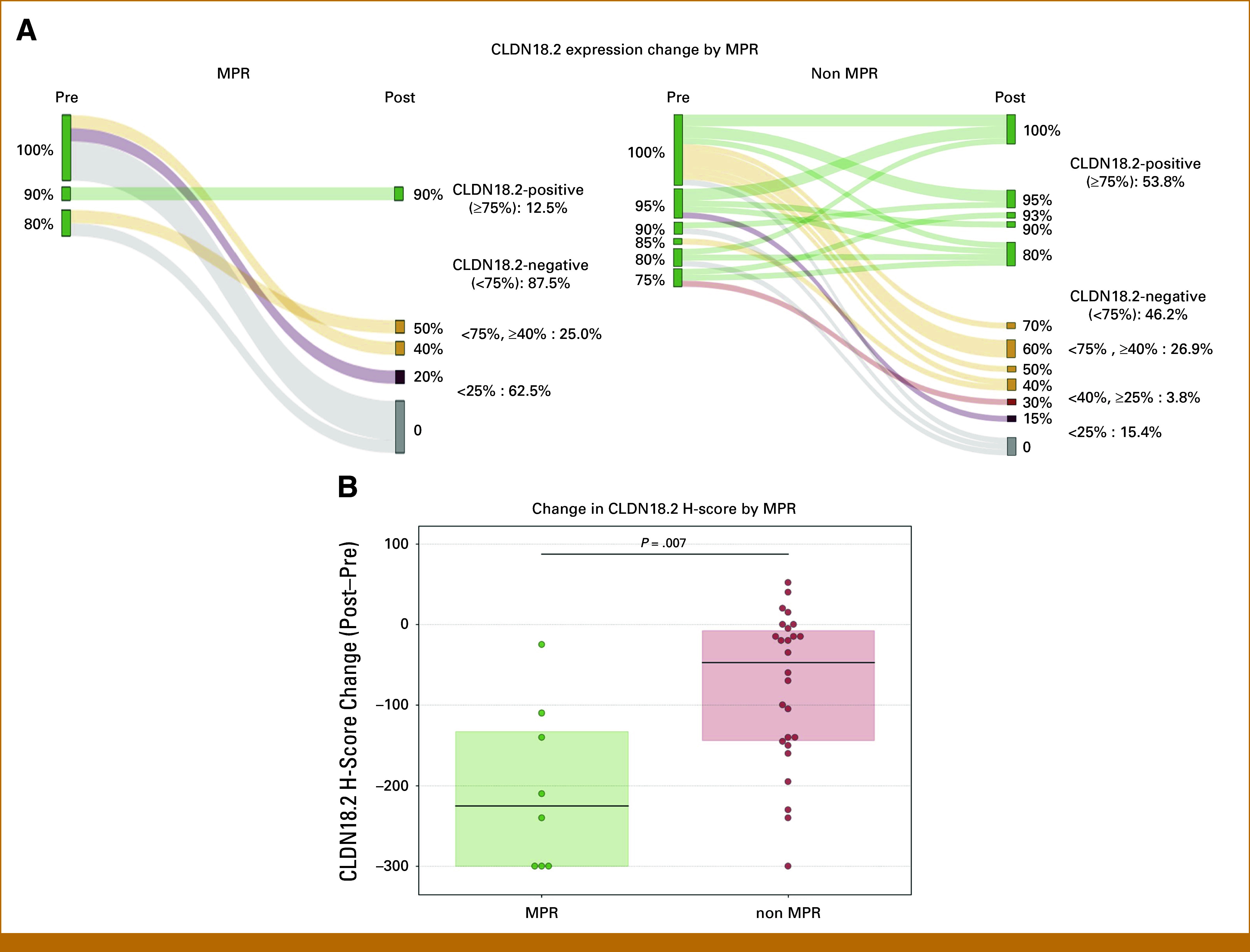
CLDN18.2 expression change by MPR. (A) Sankey diagrams comparing CLDN18.2 expression changes between MPR and non-MPR cases. In MPR cases, the CLDN18.2 positivity rates decreased to 12.5% aftertreatment, with the remaining cases subdivided into <75%, ≥40% (25.0%), and <25% (62.5%). In non-MPR cases, 53.8% remained CLDN18.2-positive, whereas 46.2% became negative, subdivided into <75%, ≥40% (26.9%); <40%, ≥25% (3.8%); and <25% (15.4%). (B) Bee-swarm plot depicting the changes in CLDN18.2 H-scores (calculated as post-treatment minus pretreatment values) stratified by the MPR status. The median H-score change was −225.0 in the MPR group compared with −47.5 in the non-MPR group (difference: −177.5). The difference between the groups was statistically significant (Mann-Whitney U test, *P* = .007). CLDN18.2, claudin-18 isoform 2; MPR, major pathological response.

**FIG A2.**
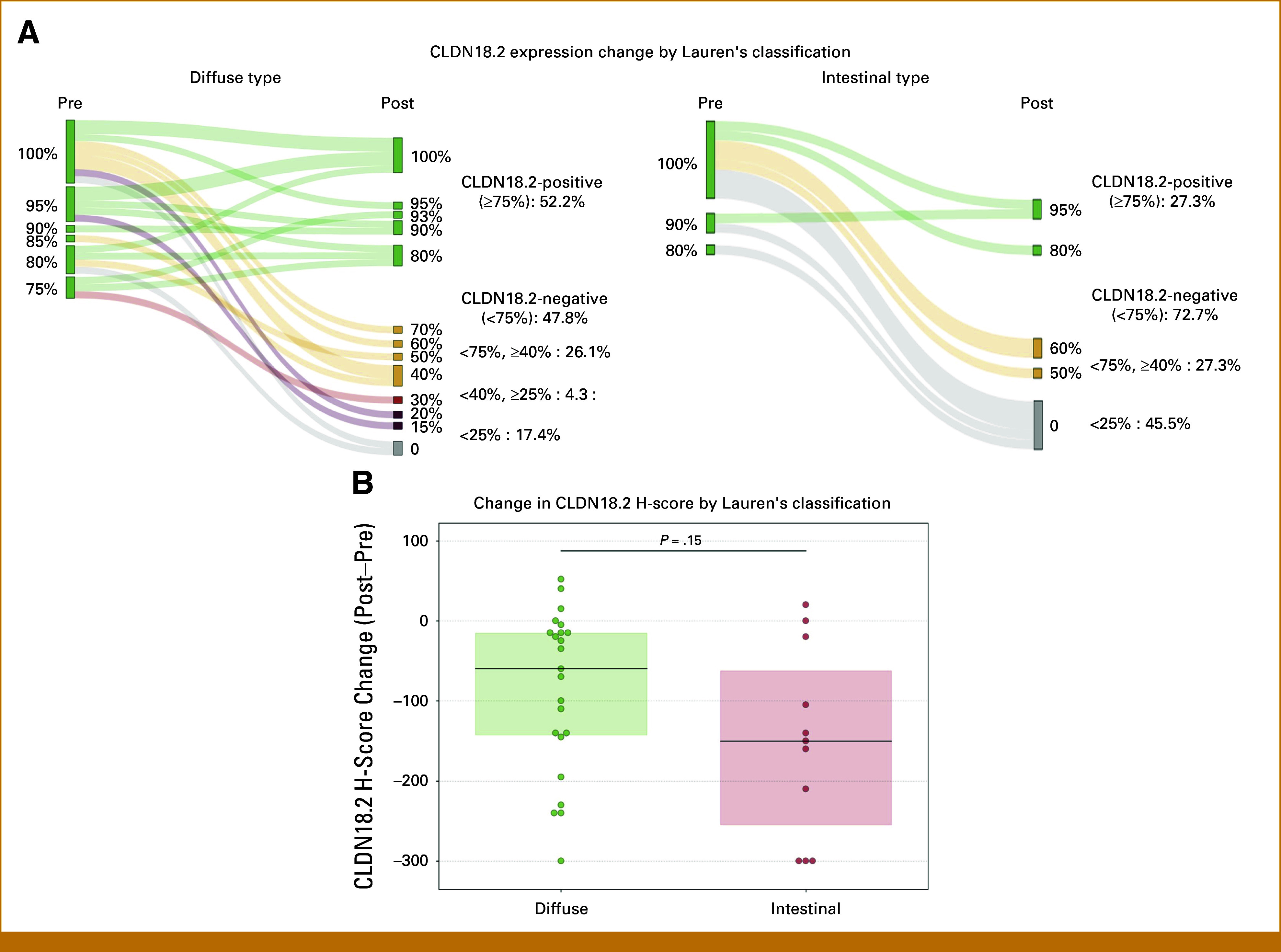
CLDN18.2 expression change by Lauren's classification. (A) Sankey diagrams comparing CLDN18.2 expression changes between the diffuse and intestinal types. In diffuse-type tumors, 52.2% remained CLDN18.2-positive after treatment, whereas 47.8% became negative, subdivided into <75%, ≥40% (26.1%); <40%, ≥25% (4.3%); and <25% (17.4%). In intestinal-type tumors, only 27.3% remained positive, with 72.7% becoming negative, subdivided into <75%, ≥40% (27.3%), and <25% (45.5%). (B) Bee-swarm plot depicting the changes in CLDN18.2 H-scores (calculated as post-treatment minus pretreatment values) stratified by Lauren's classification. The median H-score change was −65.0 in the diffuse-type compared with −150.0 in the intestinal-type tumors. The difference between the groups was not statistically significant (Mann-Whitney U test, *P* = .15). CLDN18.2, claudin-18 isoform 2.

CLDN18.2 positivity rates in resected specimens were significantly higher in patients who did not achieve MPR compared with those who achieved MPR (53.8% *v* 12.5%, *P* = .05; Fig 4A). Similarly, the decrease in H-scores was more pronounced in patients with MPR compared with those without MPR (median change −220.0 *v* −45.0, *P* = .007; Fig 4B). In addition, a trend toward a greater H-score decrease in intestinal-type histology was noted compared with diffuse-type histology (median change −150.0 *v* −65.0, *P* = .15; Appendix Fig A2).

## DISCUSSION

We evaluated the associations between clinicopathological indicators, including HER2, MMR, PD-L1, and CLDN18.2, and pathological responses in patients with resectable GC/GEJC who received perioperative FLOT. The positivity rates for HER2, dMMR, EBV, and CLDN18.2 in this population were comparable with those observed in a metastatic population,^26^ although HER2 positivity was relatively lower. This discrepancy may be due to the enrollment of several patients in HER2-directed perioperative trials during the same period.^27^ Although HER2, PD-L1, and CLDN18.2 status did not significantly influence pathological responses to preoperative FLOT, the lower MPR rate observed in diffuse histology aligns with previous studies.^28^ dMMR showed poor pathological response, which is consistent with previous reports.^7^ Notably, CLDN18.2 expression decreased significantly in resected samples, and the CLDN18.2 positivity rate was significantly lower in patients who achieved MPR than in those who did not.

Currently, limited data are available on the predictive value of HER2 and PD-L1 in patients with gastric cancer receiving perioperative FLOT. On the basis of indirect comparisons across several trials, there seems to be no remarkable difference in pathological response according to HER2 status.^17,18^ Similarly, the FLOT arm of the DANTE trial reported comparable MPR rates between the overall population (39%) and CPS ≥10 group (38%), suggesting that PD-L1 expression does not predict responses to perioperative chemotherapy.^19^ However, PD-L1 status remains relevant for predicting the additional benefit of PD-1 inhibitors in the perioperative setting.^8,19,20^

The CLDN18.2 positivity rate in our cohort was almost similar to that reported in a metastatic population.^29,30^ In patients with unresectable advanced GC/GEJC, CLDN18.2 expression is not associated with the response rate of first-line chemotherapy.^29^ Similarly, in this study, the MPR rate was comparable between CLDN18.2-positive and CLDN18.2-negative tumors (25.7% *v* 21.0%), suggesting that CLDN18.2 expression per se does not significantly affect chemotherapy efficacy. However, we observed a trend toward decreased CLDN18.2 expression following preoperative FLOT in patients with CLDN18.2-positive GC/GEJC. This reduction in CLDN18.2 expression in a subset of patients aligns with previous findings in metastatic disease, where approximately 40% of patients demonstrated decreased CLDN18.2 levels after chemotherapy.^29-31^ Although we only assessed changes in CLDN18.2 status in patients with CLDN18.2-positive GC/GEJC at baseline, upregulated CLDN18.2 expression following chemotherapy has been rarely reported in previous studies of metastatic disease.^29-31^

Notably, this study showed that a higher number of CLDN18.2-expressing tumor cells remained in resected specimens in non-MPR cases. Moreover, CLDN18.2-expressing tumor cells were more frequently observed in diffuse-type tumors. Although the exact mechanisms underlying these phenomena require further investigation, this finding raises the possibility that incorporating CLDN18.2-targeted agents into FLOT regimens could improve the pathological response. Currently, a clinical trial investigating the combination of zolbetuximab with FLOT in CLDN18.2-positive resectable GC/GEJC is underway (ClinicalTrials.gov identifier: NCT03505320).

This study has several limitations. First, its single-center design in Japan may limit the generalizability of the findings. Second, due to its retrospective nature, all reported *P* values should be considered nominal and not definitive. Furthermore, the small sample sizes in certain subgroups complicate the interpretation and necessitate caution when drawing conclusions. Additionally, we exclusively analyzed changes in CLDN18.2 expression without examining other biomarkers, focusing only on patients with high baseline expression. Finally, event-free survival and overall survival were not assessed due to the short follow-up period.

In conclusion, HER2, PD-L1 CPS, and CLDN18.2 status did not affect the pathological response to preoperative FLOT. Diffuse-type histology was a negative predictive indicator for preoperative FLOT in locally advanced GC/GEJC. CLDN18.2-expressing tumor cells were more likely to persist in patients who did not achieve MPR, suggesting that CLDN18.2-targeted therapy could be investigated as a potential strategy in patients with resectable CLDN18.2-positive GC/GEJC who do not respond adequately to FLOT alone.

## Data Availability

A data sharing statement provided by the authors is available with this article at DOI https://doi.org/10.1200/PO-25-00925. The datasets generated during and analyzed during the current study are available from the corresponding author upon reasonable request.

## APPENDIX

**TABLE A1.** Pathological Response (MPR) by Subgroup

Variables	n	MPR, n	MPR Rate, %	95% CI	Univariable Analysis		
					Odds Ratio	95% CI	*P*
Age							
≥65	81	17	21.0	12.7 to 31.5	0.77	0.30 to 1.94	.75
<65	35	9	25.7	12.5 to 43.3			
≥75	32	5	15.6	5.3 to 32.8	0.56	0.19 to 1.63	.40
<75	84	21	25.0	16.2 to 35.6			
Sex							
Male	88	22	25.0	16.4 to 35.4	2.00	0.62 to 6.40	.30
Female	28	4	14.3	4.0 to 32.7			
ECOG PS							
0	99	24	24.2	16.2 to 33.9	2.40	0.51 to 11.26	.35
1	17	2	11.8	1.5 to 36.4			
Tumor location							
GEJ	49	11	22.4	11.8 to 36.6	1.00	0.41 to 2.43	1.00
Gastric	67	15	22.4	13.1 to 34.2			
Borrmann type							
Type 4	12	1	8.3	0.2 to 38.5	0.29	0.04 to 2.34	.29
Non–type 4	104	25	24.0	16.2 to 33.4			
Lauren's type							
Diffuse	70	10	14.3	7.1 to 24.7	0.31	0.13 to 0.77	.02
Intestinal	46	16	34.8	21.4 to 50.2			
Histology							
Sig	20	3	15.0	3.2 to 37.9	0.56	0.15 to 2.08	.56
Non-sig	96	23	24.0	15.8 to 33.7			
cT4							
T4	44	8	18.2	8.2 to 32.7	0.67	0.26 to 1.70	.53
Non-T4	72	18	25.0	15.5 to 36.6			
cN							
Positive	107	24	22.4	14.9 to 31.5	1.01	0.20 to 5.20	1.00
Negative	9	2	22.2	2.8 to 60.0			
EBV status							
Positive	4	0	0.0	0.0 to 60.2	0.41	0.02 to 8.08	.57
Negative	112	26	23.2	15.8 to 32.1			
MMR status							
Deficient	7	0	0.0	0.0 to 41.0	0.23	0.01 to 4.16	.35
Proficient	109	26	23.9	16.2 to 33.0			
HER2 status							
Positive	9	2	22.2	2.8 to 60.0	0.99	0.19 to 5.07	1.00
Negative	107	24	22.4	14.9 to 31.5			
PD-L1 status							
CPS ≥ 1	105	21	20.0	12.8 to 28.9	0.30	0.08 to 1.08	.12
CPS < 1	11	5	45.5	16.7 to 76.6			
CPS ≥ 5	51	12	23.5	12.8 to 37.5	1.12	0.47 to 2.69	.98
CPS < 5	65	14	21.5	12.3 to 33.5			
CPS ≥ 10	18	3	16.7	3.6 to 41.4	0.65	0.17 to 2.45	.76
CPS < 10	98	17	23.5	15.5 to 33.1			
CLDN18.2 status							
CLDN18.2 ≥ 75	35	9	25.7	12.5 to 43.3	1.30	0.51 to 3.30	.75
CLDN18.2 < 75	81	17	21.0	12.7 to 31.5			
CLDN18.2 ≥ 40	65	15	23.1	13.5 to 35.2	1.09	0.45 to 2.64	1.00
CLDN18.2 < 40	51	11	21.6	11.3 to 35.3			
CLDN18.2 ≥ 25	69	16	23.2	13.9 to 34.9	1.12	0.46 to 2.73	.98
CLDN18.2 < 25	47	10	21.3	10.7 to 35.7			

Abbreviations: CLDN18.2, claudin-18 isoform 2; CPS, combined positive score; EBV, Epstein-Barr virus; ECOG PS, Eastern Cooperative Oncology Group Performance Status; HER2, human epidermal growth factor receptor 2; MMR, mismatch repair.
